# The Art of Prolonging Life

**Published:** 1890-08

**Authors:** 


### THE ART OF PROLONGING LIFE.

Exercise is essential to the preservation of health; inactivity is a potent cause of wasting and degeneration. The vigor and equality of the circulation, the functions of the skin, and the aeration of the blood, are all promoted by muscular activity, which thus keeps up a proper balance and relation between the important organs of the body. In youth, the vigor of the system is often so great that if one organ be sluggish another part will make amends for the deficiency by acting vicariously, and without any consequent damage to itself. In old age, the task cannot thus be shifted from one organ to another; the work allotted to each sufficiently taxes its strength, and vicarious action cannot be performed without mischief. Hence the importance of maintaining, as far as possible, the equable action of all the bodily organs, so that the share of the vital processes assigned to each shall be properly accomplished. For this reason exercise is an important part of the conduct of life in old age; but discretion is absolutely necessary. An old man should discover by experience how much exercise he can take without exhausting his powers, and should be careful never to exceed the limit. Old persons are apt to forget that their staying powers are much less than they once were, and that, while a walk of two or three miles may prove easy and pleasurable, the addition of the return journey of similar length will seriously overtax the strength.—*Dr. Roose, in Science Monthly.*

